# Modulation of Cytochrome P450, P-glycoprotein and Pregnane X Receptor by Selected Antimalarial Herbs—Implication for Herb-Drug Interaction

**DOI:** 10.3390/molecules22122049

**Published:** 2017-11-23

**Authors:** Pius S. Fasinu, Vamshi K. Manda, Olivia R. Dale, Nosa O. Egiebor, Larry A. Walker, Shabana I. Khan

**Affiliations:** 1 Department of Pharmaceutical Sciences, College of Pharmacy and Health Sciences, Campbell University, Buies Creek, NC 27506, USA; 2 National Center for Natural Products Research, School of Pharmacy, University of Mississippi, Oxford, MS 38677, USA; mandavamshi@gmail.com (V.K.M.); ordale@olemiss.edu (O.R.D.); lwalker@olemiss.edu (L.A.W.); skhan@olemiss.edu (S.I.K.); 3 Department of Environmental Resources Engineering, State University of New York College of Environmental Science and Forestry, Syracuse, NY 13210, USA; egiebor@gmail.com; 4 Department of BioMolecular Sciences, School of Pharmacy, University of Mississippi, Oxford, MS 38677, USA

**Keywords:** cytochrome P450, drug metabolism, enzyme induction, herb-drug interaction, herbal medicine, malaria, P-glycoprotein, pregnane-X factor

## Abstract

Seven medicinal plants popularly used for treating malaria in West Africa were selected to assess herb-drug interaction potential through a series of in vitro methods. Fluorescent cytochrome P450 (CYP) assays were conducted using the recombinant CYP enzymes for CYP1A2, CYP2A6, CYP2B6, CYP2C9, CYP2C19, CYP2D6 and CYP3A4 to assess the effect of the methanolic extracts on the metabolic activity of CYPs. Secondly, the inhibitory effect of the extracts was evaluated on P-glycoproteins (P-gp) using calcein-AM, a fluorescent substrate, in MDCK-II and hMDR1-MDCK-II cells. The inhibition of P-gp activity was determined as a reflection of increase in calcein-AM uptake. Additionally, the enzyme induction potential of the extracts was assessed through the modulation of PXR activity in HepG2 cells transiently transfected with pSG5-PXR and PCR5 plasmid DNA. Significant inhibition of CYP activity (*IC*_50_ < 10 µg/mL) was observed with the following herbs: *A. muricata* [CYP2C9, 3A4 and CYP2D6]; *M. indica* [CYP2C9]; *M. charantia* [CYP2C9 and CYP2C19]; *P. amarus* [CYP2C19, CYP2C9 and CYP3A4]; *T. diversifolia* [CYP2C19 and CYP3A4]. Extracts of four herbs (*P. amarus*, *M. charantia*, *T. diversifolia* and *A. muricata*) exhibited significant inhibition of P-gp with *IC*_50_ values (µg/mL) of 17 ± 1, 16 ± 0.4, 26 ± 1, and 24 ± 1, respectively. In addition, four herbs (*A. mexicana*, *M. charantia*, *P. amarus* and *T. diversifolia*) showed a >two-fold increase in induction in PXR activity. These findings suggest that these herbs may be capable of eliciting herb-drug interactions if consumed in high quantities with concomitant use of conventional therapies.

## 1. Introduction

Malaria remains one of the leading causes of death in several countries of the world. According to the 2014 malaria statistics of the World Health Organization (WHO), there is ongoing malaria transmission in 97 countries and territories around the world, with 3.3 billion people at risk of infection [1]. Artemisinin-based combination therapy (ACT) has been a mainstay of treatment, as recommended by the WHO. In resource-poor countries, there is still heavy reliance on traditional herbal remedies for the treatment of malaria [2]. Additionally, the widespread combination of local herbal preparations and antimalarial drugs has been reported [3]. Herb-drug interaction (HDI) is the most important clinical risk in concomitant herb-drug administrations. HDIs are often pharmacokinetic in nature, in which case, the presence of the herbal products alters the metabolism and/or transport of the co-administered drug. Pharmacokinetic HDIs pose more risk to the drugs that have narrow therapeutic windows [4]. Two extremes of clinical experience are possible—therapy failure due to enzyme induction that leads to faster drugs clearance, and toxicity as a result of enzyme inhibition and drug accumulation.

The modulation of major drug metabolizing enzymes, especially cytochrome P450 (CYPs) and drug transporters such as P-glycoproteins (P-gp), is responsible for most clinically relevant pharmacokinetic HDI [4,5]. Just like drugs, several herbal products have been shown to be capable of inhibiting and/or inducing various CYP isoforms and drug transporters. HDI is particularly undesirable for ACT in malaria therapy, because artemisinins, as well as some other antimalarial drugs, are prodrugs whose conversion to active metabolites is dependent on CYP [6]. The inhibition of CYP activity by concomitantly administered herbal products will predispose to therapy failure in malaria treatment. In addition to the associated morbidity and mortality of such treatment failure, the risk of resistance to ACT by malaria parasites increases upon sub-therapeutic exposure of the parasites to ACTs. One of the integral parts of the drug development process is the evaluation of the potential of a new chemical entity for the potential to cause drug-drug interaction. Similar methodologies, including the in vitro use of liver enzymes and cell lines, are now employed to evaluate HDI [7]. The results obtained from such in vitro studies can be employed to predict in vivo and clinical relevance, and the need for further study.

In this study, seven medicinal herbs used in the treatment of malaria in West Africa were selected for HDI studies. These include *Annona muricata*, *Argermone mexicana*, *Kalanchoe pinnata*, *Mangifera indica*, *Momordica charantia*, *Phyllanthus amarus* and *Tithonia diversifolia.*

*Annona muricata* Linn. (Annonaceae), known for its edible fruit, is a deciduous terrestrial plant native to Central America, and is widely found in West Africa and other tropical areas. Infusion of its leaves is traditionally used in the treatment of malaria and other parasitic infections in West Africa [8,9]. Several phytochemical compounds, including annocacin, muricatacin and isoquinoline alkaloids, have been isolated from the different morphological parts of *A. muricata* [10,11]. Crude extracts of the leaves of *A. muricata* have shown strong in vitro anti-plasmodial and anti-leishmanial activity [12,13]. 

*Argermone mexicana* (Papaveraceae), known as Mexican poppy, is a shrub found naturally in several countries. Its extracts and decoctions are used as traditional antimalarial remedies in West Africa. Major compounds isolated that are associated with its antimalarial activity include allocryptopine, berberine and protopine. Nematicidal compounds, phenolics, and several other alkaloids have also been isolated, but have not been tested against malaria [14,15]. In a prospective and dose-escalating clinical trial conducted in Mali in 80 human subjects who had symptoms of malaria (and *P. falciparum* parasitaemia >2000/µL), decoctions of *A. mexicana* showed a 73% adequate clinical response (ACR) with high tolerability and only minor side effects [16]. In a related comparative study in 301 patients with uncomplicated malaria, it was found that, after 28 days, second-line antimalarial treatment was not required for 89% of the patients who took *A. mexicana*, compared to 95% for the artesunate-amodiaquine group [17]. These findings have led to official approval of the decoctions of *A. mexicana* for the treatment of malaria in Mali, which in most cases are still taken alongside prescription antimalarial drugs [18].

*Kalanchoe pinnata* Lam (Crassulaceae) is native throughout Africa, where its leafy preparations are traditionally used to treat fever and malaria infection, although the anti-malarial principles have not been definitely determined [19,20]. The extracts of the leaves and bark of *Mangifera indica* (Anacardiaceae) are a popular traditional antimalarial remedy in West Africa. The extracts have been shown to possess a schizontocidal effect during early infection, and have also demonstrated repository activity [21]. *Momordica charantia* (Cucurbitaceae) is widely used in the treatment of malaria in West Africa. It has shown weak anti-plasmodial activity [22]. Exracts of *Phyllanthus amarus* and *Tithonia diversifolia* are also popular in West Africa for the treatment of malaria and other tropical diseases [23,24].

With the widespread use of these herbal products in malaria treatment along with the WHO-backed ACT roll-out, it is estimated that a significant portion of the population continues to use both the traditional remedies and ACTs together. The aim of the current study was, therefore, to evaluate the selected medicinal plants traditionally used for malarial treatment in terms of their HDI potential by investigating their effect on drug metabolizing enzymes (CYPs) and major efflux transporter (P-gp). In this study, methanolic extracts of the selected herbs were used because of the solubility of most phytochemicals in methanol, compared to water.

## 2. Results and Discussion

### 2.1. Inhibition of CYPs by the Herbal Extracts

**Figure 1:**
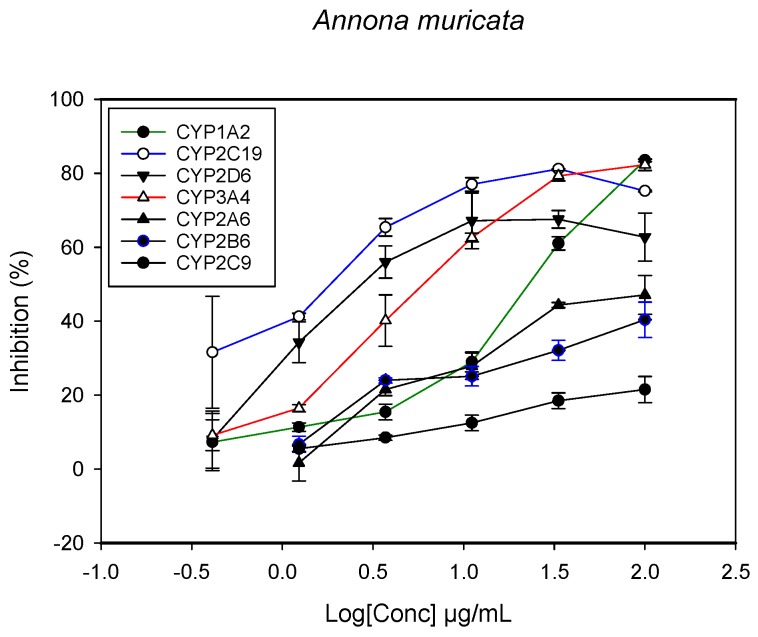
The inhibitory effect of graded concentrations of the extracts of *A. muricata* on the metabolic activity of cytochrome P450 enzymes.

**Figure 2:**
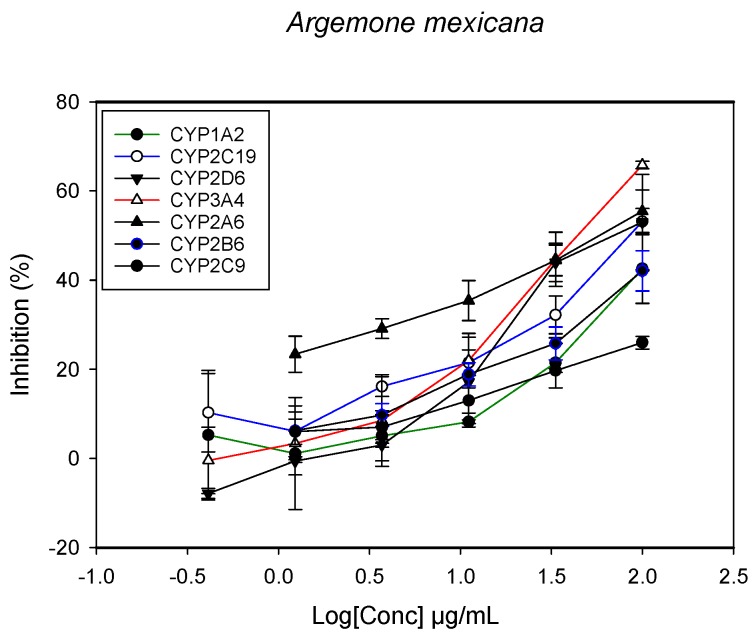
The inhibitory effect of graded concentrations of the extracts of *A. mexicana* on the metabolic activity of cytochrome P450 enzymes.

**Figure 3:**
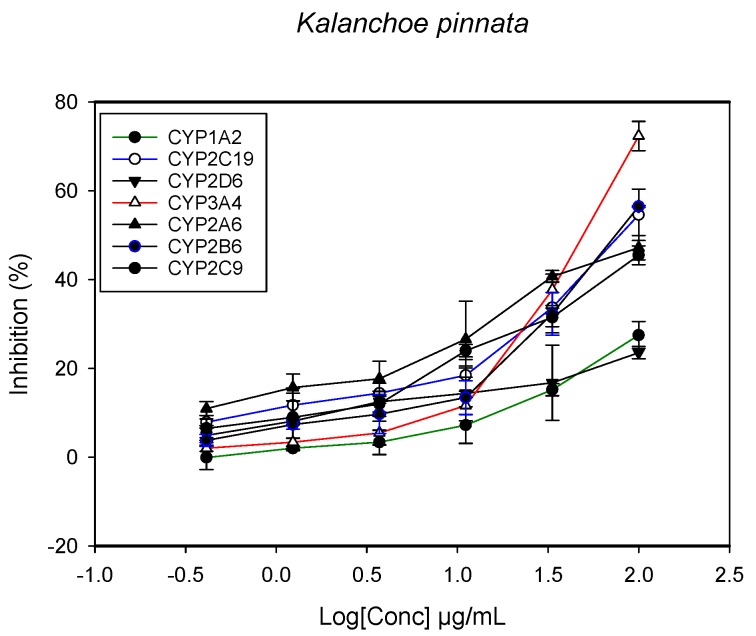
The inhibitory effect of graded concentrations of the extracts of *K. pinnata* on the metabolic activity of cytochrome P450 enzymes.

**Figure 4:**
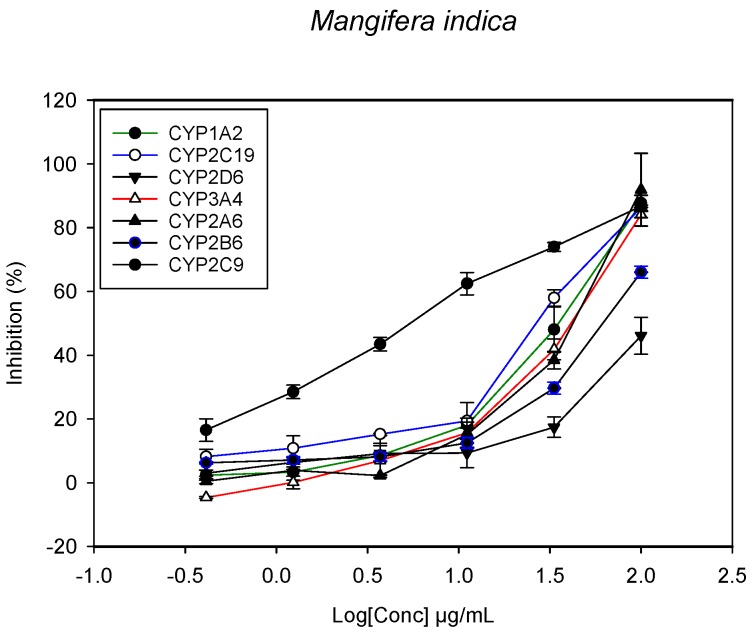
The inhibitory effect of graded concentrations of the extracts of *M. indica* on the metabolic activity of cytochrome P450 enzymes.

**Figure 5:**
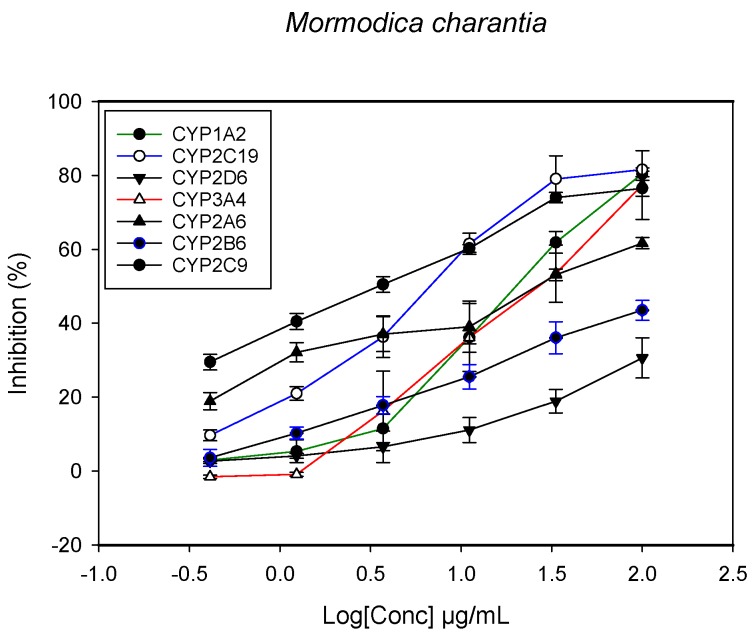
The inhibitory effect of graded concentrations of the extracts of *M. charantia* on the metabolic activity of cytochrome P450 enzymes.

**Figure 6:**
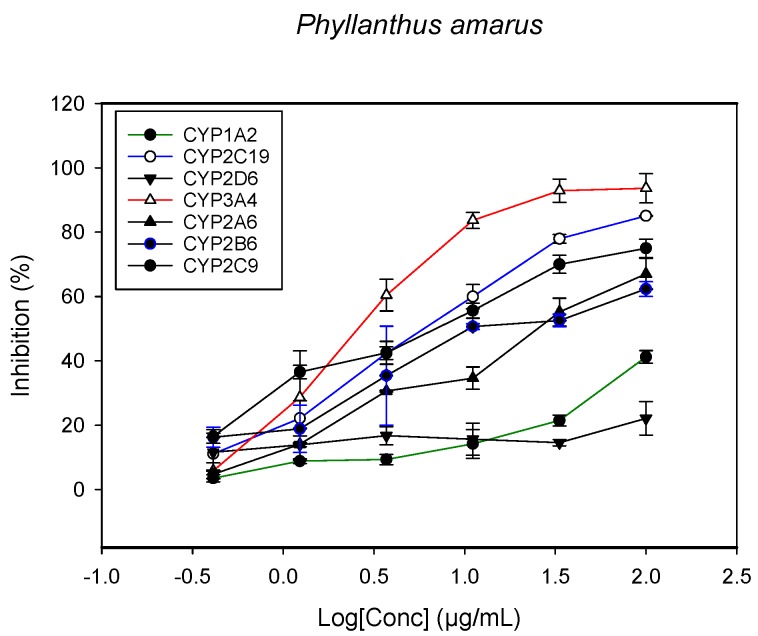
The inhibitory effect of graded concentrations of the extracts of *P. amarus* on the metabolic activity of cytochrome P450 enzymes.

**Figure 7:**
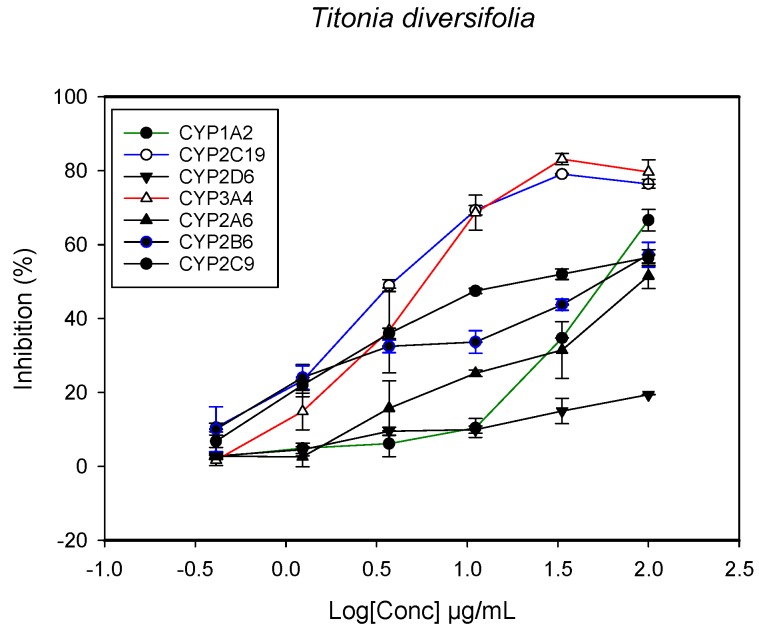
The inhibitory effect of graded concentrations of the extracts of *T. diversifolia* on the metabolic activity of cytochrome P450 enzymes.

A fluorescent CYP assay was conducted using the recombinant CYP test kits for CYP1A2, CYP2A6, CYP2B6, CYP2C9, CYP2C19, CYP2D6 and CYP3A4. The antimalarial herbs exerted inhibitory effects on the CYP isozymes to varying degrees (with *IC*_50_ values ranging from 2 to >100 µg/mL) (Figure 1, Figure 2, Figure 3, Figure 4, Figure 5, Figure 6 and Figure 7). The inhibition of CYP3A4 was the most pronounced, as all the seven herbs exerted potent inhibitory activity against this isoform. CYP2D6 was the least inhibited; only *A. muricata* and *A. Mexicana* had any activity against CYP2D6. The computed *IC*_50_ values (Table 1) showed the potency of the extracts against CYP activity. Low *IC*_50_ indicates higher potency of inhibition against the CYPs. *A. muricata* had the highest potency for inhibition of CYP2D6, CYP2C19, CYP3A4 and CYP1A2. *A. mexicana* displayed only moderate inhibition of CYP3A4, CYP2A6, CYP2D6 and CYP2C19. *K pinnata* inhibited CYP3A4, CYP2C19 and CYP2B6. *M. indica* showed inhibitory activity against all of the tested CYP isoforms, demonstrating the most potent inhibition against CYP2C9, and the least against CYP2D6. *M. charantia* strongly inhibited CYP2C9, CYP2C19, CYP1A2, CYP3A4 and CYP2A6 in that (decreasing) order. *P. amarus* had a potent inhibitory effect on all of the enzymes except CYP2D6. Its activity against CYP3A4, CYP2A6 and CYP2B6 was the most potent of the extracts. This is similar to *T. diversifolia*, which inhibited CYP1A2, CYP2C9, CYP2C19 and CYP3A4 strongly, exerted a mild inhibition effect on CYP2B6 and CYP2A6, and showed no activity against CYP2D6.

### 2.2. P-glycoprotein Inhibition

**Figure 8:**
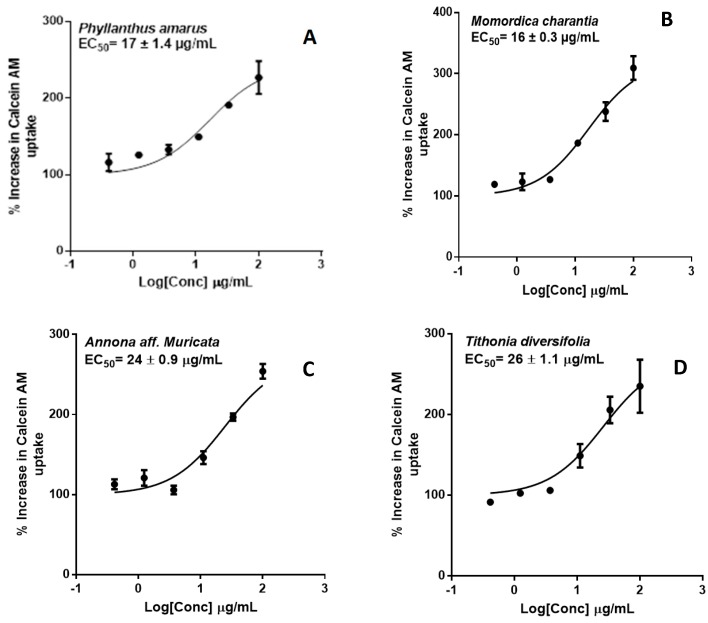

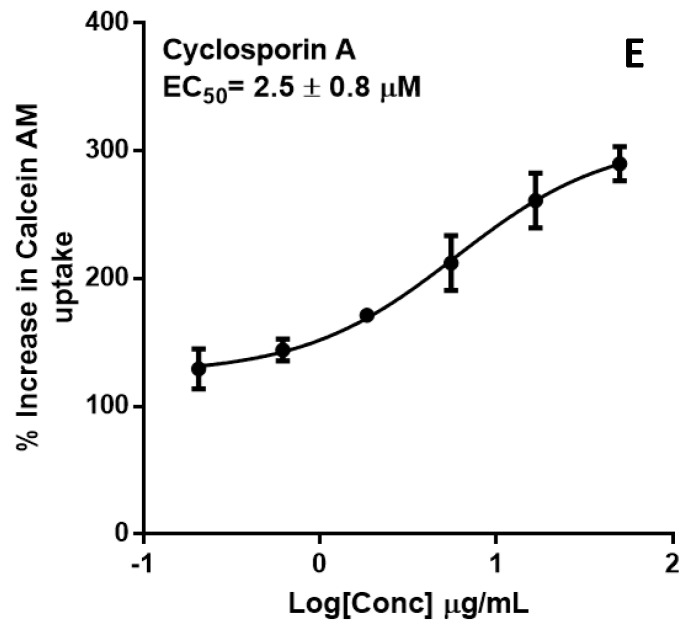
Dose-response curves of P-gp inhibition determined by calculating the percent uptake of calcein-AM into hMDR1-MDCKII cells in the presence of (**A**) *P. amarus*; (**B**) *M. charantia*; (**C**) *A. muricata*; (**D**) *T*. *diversifolia* and (**E**) cyclosporin A, the positive control.

P-gp inhibition was measured by monitoring calcein-AM uptake in transfected hMDR1-MDCKII cells. Extract concentrations were profiled against % increase in Calcein AM uptake (Figure 8). Extracts of four of the herbs—*P. amarus*, *M. charantia*, *T. diversifolia* and *A. Muricata*—exhibited significant P-gp inhibition with *EC*_50_ values (µg/mL) of 17 ± 1.4, 16 ± 0.3, 26 ± 1.1, 24 ± 0.9, respectively. These findings suggest that the herbal products, if taken in sufficient quantities, may inhibit the activity of intestinal P-gp and alter the absorption kinetics of its substrates. 

### 2.3. Pregnane-X Receptor Modulation 

The effects of the methanolic extracts of seven antimalarial herbs on PXR were assessed in HepG2 cells. PXR-transfected HepG2 cells were treated with the extracts for 24 h, and the fold increase in luciferase activity was measured as an index of PXR activation. A fold-increase in induction of 2 or more in the PXR activity is considered significant (indicating a 100% increase compared to vehicle control) [25]. Cells treated with methanol and rifampicin served as vehicle and positive controls, respectively.

As shown in Table 2, three extracts (A. *mexicana*, *M. charantia*, and *P. amarus)* showed >2-fold increase in induction in PXR activity at a concentration of 20 µg/mL. *P. amarus* and *M. charantia* exhibited the strongest activity (>4 fold) at 60 µg/mL. However, *T. diversifolia* showed maximal activity (2.4 fold induction) at a lower concentration (6.67 µg/mL). These results indicate that these four extracts may significantly modulate the transcriptional activity of PXR, and could thereby affect the expression of downstream genes involved in PXR signaling. Additionally, no cytotoxicity was observed toward HepG2 cells. 

### 2.4. Discussion

HDI studies have gained prominence in recent times due to the increasing clinical reports of herbal products interfering with the effects of drugs concurrently consumed. Failure of contraceptives due to the inductive effect of St John’s wort or bleeding from warfarin therapy due to the inhibition of warfarin metabolism by ginkgo are documented examples of clinically significant HDI [26,27]. Not much has been studied in terms of the effect of herbal products on anti-infective drugs. In malaria treatment especially, where the utility of existing drugs is threatened by resistant strains of the parasite, subtherapeutic exposure to the drugs is of great concern. This is particularly so considering that the major antimalarial drugs—artemisinins (artesunate, arteeter, arthemete), amodiaquine and primaquine—are prodrugs whose conversion to active metabolites is CYP-dependent. The inhibition of CYP activity is thus not desirable when these antimalarial drugs are used. 

The modulatory activity of artemisinins themselves on drug-metabolizing enzymes have been reported in previous studies. Artemisinins undergo CYP2B6-medicated autoinduction, an underlying mechanism that has been used to explain the observed high recrudescence rate associated with its antimalarial use [28,29,30]. The molecular mechanism for the interaction of artemisinins with drug-metabolizing enzymes is its ability to activate PXR and constitutive androstane receptor (CAR). Thus, drug interactions with artemisinins have been a concern in malaria treatment due to the fact that most combination drugs are substrates of CYPs [31]. The concurrent use of herbal products will further increase this risk.

In the present study, several herbs exhibited potent inhibitory effects on CYP2A6, CYP2B6 and 3A4, which are the major isoforms involved in the formation of the active dihydroartemisinin from the artemisinins. The inhibition of the CYP2C family by all the extracts is also instructive, as this enzyme family metabolizes amodiaquine to its active metabolite [32]. However, the extent of in vivo absorption of these herbs is not known, and the current findings suggest that the concomitant use of the herbs and the antimalarial drugs may be undesirable. The overall bioavailability could be affected due to the presence of CYP and P-gp in the intestinal mucosa. It may thus be advisable to avoid concurrent consumption of antimalarial drugs and the implicated antimalarial herbs, in order to avoid the suppression of the formation of the active metabolites.

P-gp is an important mediator of HDI. In vitro inhibition/induction of P-gp is an important indicator for HDI because of the abundant expression of P-gp in the intestinal epithelium. This implies that, regardless of absorption into the systemic circulation, P-gp inhibition can alter the bioavailability of orally administered drugs. Calcein-AM is a commonly used probe substrates of P-gp [33]. In the calcein-AM assay, *A. muricata*, *M. charantia*, *P. amarus* and *T. diversifolia* exhibited significant P-gp inhibition similar to cyclosporin A, the positive control (Figure 8).

Apart from enzyme/transporter inhibition, the induction of metabolic enzymes/transporters has been recognized as a common mechanism of HDI. The use of the human PXR-transfected HepG2 cells as an in vitro tool for evaluating the potential for clinically significant induction of drug-metabolizing enzymes and transporter proteins is well established [34]. Herbal products including St John’s wort and ginkgo, known to induce metabolic enzymes and transporter proteins have been shown to be activators of PXR [35,36]. The ability of *A. mexicana*, *M. charantia*, *P. amarus*, and *T. diversifolia* to cause more than a two-fold increase in PXR activity is an indication that they may cause an increased expression of drug metabolizing enzymes and/or transporter, which is considered a significant factor that causes HDI [37]. Induction of enzyme activity is usually undesirable, due to increased drug metabolism and the risk of sub-therapeutic exposure of infective organism to the drug. It is also a risk for the development of drug resistance. 

Further studies, including the determination of the extent of absorption of the active phytochemicals in the herbs and their interactions in humans, may provide more useful information which would be helpful in establishing the clinical significance of these findings.

## 3. Materials and Methods

### 3.1. Materials

Methanolic extracts of the seven selected herbs (prepared from voucher specimens) were obtained from the repository of the National Center for Natural Products Research (NCNPR), School of Pharmacy, University of Mississippi. CYP1A2/CEC, CYP2C9/MFC, CYP2C19/CEC, CYP2D6/AMMC and CYP3A4/BQ high throughput inhibitor screening kits (containing recombinant human CYPs, substrates and relevant buffers systems) were purchased from BD Gentest (Woburn, MA, USA). Transwell plates (12 mm diameter, 0.4 μM pore size) were purchased from Costar Corp. (Cambridge, MA, USA). Madin-Darby canine kidney-II (parental) and hMDR1-MDCKII (transfected) cell lines were a gift from Dr. Gottesman (NIH, Bethesda, MD, USA). Fetal bovine serum (FBS) was purchased from Hyclone Lab Inc. (Logan, UT, USA). Dulbecco’s Modified Eagle Medium (DMEM), Minimal Essential Medium (MEM), Hanks balanced salt solution (HBSS), HEPES, Trypsin EDTA, Penicillin streptomycin, and Sodium Pyruvate were purchased from GIBCO BRL (Invitrogen Corp., Grand Island, NY, USA). Troleandomycin was from Santa Cruz Biotechnology, Inc. (Dallas, TX, USA). All other chemicals were from Sigma Chem. Co., (St. Louis, MO, USA). 

### 3.2. Inhibition Assay with Recombinant CYPs 

The assay to determine the inhibitory activity of the selected medicinal plants on the metabolic activity of CYPs was conducted under similar conditions as earlier reported [38,39]. The extracts and positive controls were serially diluted in a solution (100 μL) of cofactors mix, CYP proteins (0.05 mg of protein/mL), and G-6-PDH to achieve six concentrations ranging from 0.4 to 100 µg/mL. Methanol was used as the negative control. Initial readings were taken to record any inherent fluorescence and the plates were incubated at 37 °C for 10 min. Enzymatic reaction was initiated by the addition of the enzyme substrate mixture (100 μL) followed by incubation for 15, 30, or 45 min (according to the supplier instructions for each enzyme). The total volume of the incubation mixture was 200 μL in 96-well microplates. The reaction was terminated by the addition of 75 μL of ice cold acetonitrile/0.5 M Tris base (80:20). Fluorescence was measured on a Spectramax M5 plate reader (Molecular Devices, Sunnyvale, CA, USA) at specified excitation and emission wavelengths for each substrate. The tested concentrations were profiled against observed enzyme inhibition (%) in order to obtain the *IC*_50_ values of each extract.

### 3.3. Assay for P-glycoprotein Inhibition by Calcein-AM Uptake in Parental and Transfected MDCK-II Cells 

In order to determine the influence of the extracts on the efflux activity of P-gp, a similar assay was performed to that previously described [33]. Typically, cells were seeded in 96-well plates at about 70,000 cells/well in 200 μL of culture medium. The medium was changed 24 h after seeding and the assay was performed 48 h later. Varying concentrations (0.4–100 μg/mL) of the herbal extracts and the positive control (cyclosporine A 0.4–100 μM) were added to the cells in 50 μL of transport buffer and incubated at 37 °C for 10 min. Methanol was used as the negative control. Calcein-AM (1 μM), a fluorescent substrate of P-gp, was added and the plates were immediately placed on Spectramax and fluorescence was read up to 1 h at 15-min intervals at excitation and emission wavelengths of 485 and 530 nm, respectively. Changes in calcein-AM uptake were calculated as described previously [40,41]. The concentration that causeed 50% increase in calcein-AM uptake (*IC*_50_) was determined by plotting the increase (%) in calcein-AM uptake against the log concentration using GraphPad Prism software V.7.0 (GraphPad Software, Inc., San Diego, CA, USA)

### 3.4. Assay for PXR Modulation 

The PXR modulation assay was performed as previously described [42]. The modulation of PXR activity by the selected extracts was determined in HepG2 cells transiently transfected with pSG5-PXR (25 μg) and PCR5 plasmid DNA (25 μg) by electroporation at 180 V, 1 pulse for 70 ms. The cells were plated in 96-well plates at a density of 50,000 cells per well and left for 24 h before varying concentrations of extracts and control compounds (including methanol as negative control) were added. This was followed by 24-h incubation, after which the media was aspirated and 40 μL of luciferase reagent (Promega Corporation, Madison, WI, USA) was added to each well. The luminescence was then measured on Spectramax M5 plate reader (Molecular Devices, Sunnyvale, CA, USA). The fold induction in luciferase activity in the treated cells was calculated in comparison to vehicle-treated cells. The cell viability was determined using the CellTiter 96 AQueous One Solution Cell Proliferation Assay (MTS), as described previously as a measure of the cytotoxic effect of the extracts on the HepG2 cells [39].

### 3.5. Calculation of IC*_50_* Inhibition Values

The activity (100%) of the enzymes/cells was defined in terms of metabolite production/substrate uptake in the absence of inhibitor (negative controls). The enzyme/calceine uptake inhibition parameter (*IC*_50_) was calculated by using the kinetic equation for sigmoid curves (Equation (1)), where *x* = concentration, *y* = relative enzyme activity, and *s* = slope factor.

(1) $$y=\;\frac{100\%}{1+\;{\left(\begin{array}{c} x\\ IC50 \end{array}\right)}^{S}}$$

### 3.6. Statistical Methods 

All determinations were performed in triplicate, and values are represented as mean ± SD (*n* = 3). Statistical analysis was performed using one-way ANOVA and Dunnett’s multiple comparison tests with GraphPad Prism Version 5 (San Diego, CA, USA). *p* < 0.05 was considered to be statistically significant.

## 4. Conclusions

The findings from this study have demonstrated that the methanolic extracts of the following herbs inhibit the indicated CYP enzymes: *A. muricata*—CYP2C9, 3A4 and CYP2D6; *M. indica*—CYP2C9; *M. charantia*—CYP2C9 and CYP2C19; *P. amarus*—CYP2C19, CYP2C9 and CYP3A4; and *T. diversifolia*—CYP2C19 and CYP3A4. The extracts of *P. amarus*, *M. charantia*, *T diversifolia* and *A. muricata* exhibited significant P-gp inhibition. Additionally, *A. mexicana*, *M. charantia*, *P. amarus*, and *T. diversifolia*—showed >2-fold induction in PXR activity. By implication, the concomitant use of products containing these herbs with prescription drugs may pose a risk for HDI. Further studies are warranted in this regard.

## Figures and Tables

**Table 1:** The inhibitory potency of the selected antimalarial herbs on the activity of CYP enzymes.

Herbs (Part Used)	1A2	2A6	2B6	2C9	2C19	2D6	3A4
	*IC*_50_ (µg/mL)						
*Annona muricata* (leaves)	26.1 ± 3.1	-	>100	-	2.1 ± 1.5	1.85 ± 3.3	2.2 ± 4.4
*Argemone Mexicana* (whole plant)	>100	93.1 ± 8.5	>100	-	98.2 ± 9.3	96.1 ± 8.2	55.0 ± 5.1
*Kalanchoe pinnata* (stem)	-	>100	96.1 ± 7.7	-	91.2 ± 5.8	-	55.3 ± 7.2
*Mangifera indica* (stem bark)	42.2 ± 4.7	47.3 ± 4.1	87.4 ± 8.1	3.3 ± 0.6	32.4 ± 5.1	>100	44.2 ± 3.1
*Momordica charantia* (leaves and stem)	23.4 ± 4.3	34.1 ± 3.3	-	3.3 ± 0.5	6.4 ± 1.5	-	31.1 ± 5.2
*Phyllanthus amarus* (leaves, root and stem)	>100	28.1 ± 2.6	11.2 ± 1.5	5.3 ± 1.1	3.8 ± 1.0	-	0.5 ± 0.2
*Tithonia diversifolia* (leaves)	64.3 ± 5.2	>100	98.4 ± 5.5	21.2 ± 2.0	3.8 ± 4.5	-	3.5 ± 2.2

(-) indicates absence of inhibition.

**Table 2:** The induction of pregnane X receptor activity and the inhibition of P-glycoprotein by the extracts of 7 selected plants.

	Fold Induction in PXR Activity				
Sample/Conc (µg/mL)	60	20	6.67	2.22	0.74
*Annona muricata* (leaves)	1.66	1.17	1.14	0.89	0.89
*Argemone Mexicana* (whole plant)	2.50	2.09	1.41	1.33	1.03
*Kalanchoe pinnata* (stem)	-	-	-	-	-
*Mangifera indica* (stem bark)	-	-	-	-	-
*Momordica charantia* (leaves and stem)	4.64	2.67	1.67	1.22	1.25
*Phyllanthus amarus* (leaves, root and stem)	4.71	3.67	2.34	1.44	1.04
*Tithonia diversifolia* (leaves)	-	1.47	2.41	1.74	1.35

(-) indicates absence of induction.
